# *In ovo* Inoculation of *Bacillus subtilis* and Raffinose Affects Growth Performance, Cecal Microbiota, Volatile Fatty Acid, Ileal Morphology and Gene Expression, and Sustainability of Broiler Chickens (*Gallus gallus*)

**DOI:** 10.3389/fnut.2022.903847

**Published:** 2022-05-31

**Authors:** Abdelrazeq M. Shehata, Vinod K. Paswan, Youssef A. Attia, Mohammed Sh. Abougabal, Tarek Khamis, Amany I. Alqosaibi, Mashael M. Alnamshan, Reda Elmazoudy, Mohamed A. Abaza, Ehab A. A. Salama, Mohamed T. El-Saadony, Ahmed M. Saad, Abdel-Moneim Eid Abdel-Moneim

**Affiliations:** ^1^Department of Animal Production, Faculty of Agriculture, Al-Azhar University, Cairo, Egypt; ^2^Department of Dairy Science and Food Technology, Institute of Agricultural Sciences, Banaras Hindu University, Varanasi, India; ^3^Department of Animal and Poultry Production, Faculty of Agriculture, Damanhour University, Damanhour, Egypt; ^4^Sustainable Agriculture Research Group, Department of Agriculture, Faculty of Environmental Sciences, King Abdulaziz University, Jeddah, Saudi Arabia; ^5^Department of Pharmacology, Faculty of Veterinary Medicine, Zagazig University, Zagazig, Egypt; ^6^Laboratory of Biotechnology, Faculty of Veterinary Medicine, Zagazig University, Zagazig, Egypt; ^7^Biology Department, College of Science, Imam Abdulrahman Bin Faisal University, Dammam, Saudi Arabia; ^8^Avian and Rabbit Diseases Department, Faculty of Veterinary Medicine, Benha University, Banha, Egypt; ^9^Agricultural Botany Department, Faculty of Agriculture (Saba Basha), Alexandria University, Alexandria, Egypt; ^10^Department of Agricultural Microbiology, Faculty of Agriculture, Zagazig University, Zagazig, Egypt; ^11^Biochemistry Department, Faculty of Agriculture, Zagazig University, Zagazig, Egypt; ^12^Biological Applications Department, Nuclear Research Center, Egyptian Atomic Energy Authority, Abu Zaabal, Egypt

**Keywords:** bioactive compounds, *in ovo* feeding, gut microbiota, volatile fatty acid, ileal architecture, gene expression, sustainability, broiler chickens

## Abstract

Banning antibiotic growth promoters has negatively impacted poultry production and sustainability, which led to exploring efficient alternatives such as probiotics, probiotics, and synbiotics. Effect of *in ovo* injection of *Bacillus subtilis*, raffinose, and their synbiotics on growth performance, cecal microbial population and volatile fatty acid concentration, ileal histomorphology, and ileal gene expression was investigated in broilers (*Gallus gallus*) raised for 21 days. On 300 h of incubation, a total of 1,500 embryonated eggs were equally allotted into 10 groups. The first was non-injected (NC) and the remaining *in ovo* injected with sterile distilled water (PC), *B. subtilis* 4 × 10^5^ and 4 × 10^6^ CFU (BS1 and BS2), Raffinose 2 and 3 mg (R1 and R2), *B. subtilis* 4 × 10^5^ CFU + raffinose 2 mg (BS1R1), *B. subtilis* 4 × 10^5^ CFU + raffinose 3 mg (BS1R2), *B. subtilis* 4 × 10^6^ CFU + raffinose 2 mg (BS2R1), and *B. subtilis* 4 × 10^6^ CFU + raffinose 3 mg (BS2R2). At hatch, 60 chicks from each group were randomly chosen, divided into groups of 6 replicates (10 birds/replicate), and fed with a corn–soybean-based diet. *In ovo* inoculation of *B. subtilis* and raffinose alone or combinations significantly improved body weight, feed intake, and feed conversion ratio of 21-day-old broilers compared to NC. Cecal concentrations of butyric, pentanoic, propionic, and isobutyric acids were significantly elevated in R1, R2, BS2R1, and BS2R2, whereas isovaleric and acetic acids were significantly increased in R1 and BS2R1 compared to NC. Cecal microbial population was significantly altered in treated groups. Ileal villus height was increased (*p* < 0.001) in BS1, R2, and BS2R2 compared to NC. The mRNA expression of mucin-2 was upregulated (*p* < 0.05) in synbiotic groups except for BS1R1. Vascular endothelial growth factor (VEGF) expression was increased (*p* < 0.05) in BS2, R1, BS1R1, and BS1R2 compared to NC. SGLT-1 expression was upregulated (*p* < 0.05) in all treated birds except those of R1 group compared to NC. The mRNA expressions of interleukin (IL)-2 and toll-like receptor (TLR)-4 were downregulated (*p* < 0.05) in BS2 and R1 for IL-2 and BS1R1 and BS2R2 for TLR-4. It was concluded that *in ovo B. subtilis*, raffinose, and synbiotics positively affected growth performance, cecal microbiota, gut health, immune responses, and thus the sustainability of production in 21-day-old broilers.

## Introduction

The growing public pressure to ban sub-therapeutic antibiotics from poultry diets has impacted poultry producers by losing profits and seeking alternatives to achieve the same productivity and food quality control (1–4). Therefore, an urgent need exists to understand better the molecular and cellular interaction between the gut microbiota and host that natural compounds may manipulate to maintain gut homeostasis and enhance growth performance and animal productivity (5, 6). In chickens, the gut microbiota is critical to the host’s health, as it affects immunological responses and nutrition utilization and maintains the digestive system in proper working order (7).

Early colonization of the chicken gut by healthy bacteria provides better protection against future environmental and disease threats. The commensal gut microbiota competes with pathogens and assists the host’s intestinal epithelium and immune system maturity (8, 9). A healthy gastrointestinal system with optimal structure and function is required for broiler chickens to achieve rapid growth rates (10). Commercially, first exposure to pathogenic bacteria can occur before hatch due to hatchery or farm contamination, resulting in early chick mortality and severe economic losses (7, 11). Therefore, the early establishment of beneficial bacteria in the chicken gut is critical for preventing pathogen colonization, enhancing the immune system and gastrointestinal development, and overall health (7, 12, 13). Probiotics, prebiotics, and synbiotics are some of the compounds investigated as possible alternatives to antibiotic growth promoters in the poultry industry.

Probiotics are beneficial living bacteria that enhance innate and adaptive immunity and protect against intestinal inflammation (14). It has been found that the majority of probiotic microorganisms are Gram-positive bacteria, such as *Bacillus* spp., *Lactobacillus, Bifidobacteria* spp., and *Lactococcus* spp. (12, 15). Some probiotic bacteria are known to produce bioactive substances such as antimicrobial peptides and bacteriocins that can exert an antimicrobial effect against pathogenic and undesirable bacteria (16). The metabolic slowdown of the spore-forming *B. subtilis* helps resist severe conditions, including harsh pH and temperature conditions (17, 18). Therefore, these bacteria can benefit the host’s health *via* decreasing intestinal pH, boosting the immune system, preventing the pathogen growth, enhancing the gut development, and promoting the growth performance (19, 20).

Prebiotics are specialized plant fiber that acts as substrates for beneficial bacteria. Raffinose, as a prebiotic, is a trisaccharide compound found in whole grains, cabbage, beans, brussels sprouts, asparagus, and other vegetables. Prebiotics modulate the gut microbiota by improving the abundance of specific beneficial bacteria. Therefore, it alters the structure of the microbiota community and enhances host’s health. Furthermore, prebiotics can affect nutrient utilization, most likely through prebiotic–microbe interactions (7). Hence, combining probiotics and appropriate prebiotics (synbiotics) is an innovative and revolutionary method to collect the benefits of their biological interactions, which can improve nutrient uptake and host health (21).

Administrating bioactive compounds in poultry feed may encounter obstacles, such as exposure to the high temperature during the manufacturing process, affecting their nutritional value or bioactive functions (12). In addition, the biological value of these substances, when supplemented in drinking water, may be affected by watering devices and water quality. *In ovo* route is an innovative and effective method, especially as it delivers small amounts of bioactive substances with high efficiency compared to other supplementation routes (12, 22, 23).

Previous studies demonstrated that *in ovo* inoculation of probiotics (22, 23), prebiotics, and synbiotics (21, 24) maintained the balance of gut microbiota, improved the growth performance, and enhanced the sustainability of broiler chickens. However, limited studies have been conducted on *in ovo* injection of *B. subtilis* and their combination with raffinose in broiler chickens. Based on the above considerations, we hypothesized that *in ovo* injection of *B. subtilis*, raffinose, and their synbiotics would improve the performance and overall health status of broiler chickens at 21 days of age. Therefore, by using the *Gallus gallus in vivo* model (7, 25), the current study was conducted to evaluate the effect of *in ovo* inoculation with different levels of probiotic (*B. subtilis* PB6), prebiotic (raffinose), and their combinations on growth performance, cecal microbial population, cecal volatile fatty acid (VFA) concentration, ileal histomorphology, and ileal gene expression.

## Materials and Methods

### Ethics Statement

All animal care procedures were approved by the Central Animal Ethical Committee of Banaras Hindu University (542/GO/ReBi/S/02/CPCSEA 2017)/IAEC/3037.

### Incubation and Materials

Fertile eggs were obtained from a local broiler breeder facility (Indian River) at 48 weeks of age. Petersime incubator (Petersime Nv, Zulte, Belgium) was used for egg incubation following the standard commercial conditions (37.5°C and 60% relative humidity). Egg weight was approximately 65.7 g. On day 10 of egg incubation, the eggs were candled, and infertile, non-viable, and contaminated eggs were discarded. In this study, we have used probiotics *B. subtilis* PB6 (ClOSTAT) provided by Kemin^®^ (Herentals, Belgium) and prebiotic (Raffinose) supplied by Sigma-Aldrich (St. Louis, MO, United States).

### *In ovo* Treatment

After 300 h of incubation, a total of 1,500 embryonated eggs were randomly allotted to 10 groups (*n* = 150 embryonated eggs per group). The treatment solutions were prepared on the day of injection. Approximately 0.2 ml of treatment solutions were injected into the air cell using a 21-gauge needle on an automatic injector (NJ Phillips Pty Limited, Somersby, Australia). The applied treatments were: (1) non-injected (NC) group was non-injected; (2) sterile distilled water (PC) group was injected with sterile distilled water; (3) *in ovo* injection with *B. subtilis* 4 × 10^5^CFU/egg (BS1); (4) *in ovo* injection with *B. subtilis* 4 × 10^6^CFU/egg (BS2); (5) *in ovo* injection with Raffinose 2 mg/egg (R1); (6) *in ovo* injection with raffinose 3 mg/egg (R2); (7) *in ovo* injection with *B. subtilis* 4 × 10^5^CFU + raffinose 2 mg/egg (BS1R1); (8) *in ovo* injection with *B. subtilis* 4 × 10^5^CFU + raffinose 3 mg/egg (BS1R2); (9) *in ovo* injection with *B. subtilis* 4 × 10^6^CFU + raffinose 2 mg/egg (BS2R1); and (10) *in ovo* injection with *B. subtilis* 4 × 10^6^CFU + raffinose 3 mg/egg (BS2R2). The doses of probiotic and synbiotic were previously determined by a preliminary experiment. The effect of different concentrations of both probiotic and synbiotic on embryonic mortality and hatchability was tested. As for prebiotic, concentrations were used, according to previous research (26).

### Chicks, Diets, and Experimental Design

At hatch, 60 chicks from each treatment (600 chicks in total) were randomly chosen, allocated to 6 replicates (10 chicks each), and caged in separated metal cages (50 cm × 35 cm × 34 cm) prepared for the newly hatched chicks under controlled environmental conditions and continuous lighting. Feed and drinking water were provided *ad libitum*. Birds were fed on a crumbled diet (corn- and soybean meal-based diets) for a 21-day post-hatch trial period (starter diet, day 1 to 10; grower, day 11 to 21). Nutrient composition of diets (Table 1) was calculated based on NRC (27) tables of feedstuff analysis to meet the nutrient requirements of the strain, Indian River, Aviagen 2019. All birds were vaccinated against Newcastle disease virus (NDV) and infectious bronchitis virus (IBV) on the 7th day and given a booster against NDV on the 17th day of the experiment. Amprolium 20% (water-soluble powder), 75 g/100 L of drinking water, was used for 3 days during the second week as an anticoccidial drug.

**TABLE 1:** Composition and calculated analysis of diets.

Ingredients (%)	Starter (d 1-11)	Grower (d 12-21)
Corn 8%	52.64	54.50
Corn gluten meal 62%	4.70	4.71
Soybean meal 44%	35.09	32.70
Vegetable oil	2.91	4.00
Limestone	1.60	1.41
Monocalcium phosphate	1.70	1.55
Salt	0.30	0.30
Sodium bicarbonate	0.20	0.10
Premix^1^	0.30	0.30
L-Lysine	0.32	0.23
Dl-Methionine	0.24	0.20
Total	100.00	100.00
Calculated analysis		
ME (Kcal/kg)	3000.42	3100.11
Crude protein	23.00	22.00
Calcium	0.96	0.87
Available Phosphorus	0.48	0.435
Lysine	1.44	1.30
Methionine	0.62	0.57
Total sulfur amino acids (%)	1.00	0.93

^1^Provides per kg of diet: Vitamin A, 12,500 I.U; Vitamin D3, 4,000 I.U; Vitamin E, 20.00 IU; Vitamin K3, 4.00 mg; Vitamin B1, 4.0 mg; Vitamin B2, 6.0 mg; Vitamin B6, 5.00 mg; Vitamin B12, 20.0 mg; Niacin, 60.0 mg; D-Biotin, 200.0 mg; Calcium D-pantothenate, 18.333 mg; Folic acid, 2.083 mg; manganese, 100.0 mg; iron, 80.0 mg; ZnSO_4_⋅H_2_O, 212.52 mg; CuSO_4_⋅H_2_O, 31.18 mg; iodine, 2.0 mg; cobalt, 500.0 mg; and selenium, 250.0 mg.

**TABLE 2:** Sequences of primers used for relative real-time PCR analysis.

Gene name	Sequence of the primer	product size	GenBank accession No.
*Mucin-2 (MUC-2)*	Forward: CCAGACTGGACTTCACGGAC	129	XM_040673077.1
	Reverse: ACAGCCCCCTCTACCATCAT		
*Toll-like receptor-4 (TLR-4)*	Forward: AGGCACCTGAGCTTTTCCTC	96	NM_001030693.1
	Reverse: TACCAACGTGAGGTTGAGCC		
*Interleukin-2 (IL-2)*	Forward: CACACCGGAAGTGAATGCAA	197	NM_204153.1
	Reverse: AGCAGATTAGTTAGCCACGGG		
*Na* + */glucose co-transporter-1 (SGLT-1)*	Forward: TTCTTTCTGGCTGGACGGAG	87	NM_001293240.1
	Reverse: GCCCACAAAATGTCCACTGC		
*Excitatory amino acid transporter-3 (EAAT-3)*	Forward: GGGAAGATTGGTTTGCGAGC	170	XM_424930.7
	Reverse: TCCAGCATGGCATCAACAGT		
*Vascular endothelial growth factor (VEGF)*	Forward: AGTCAGCACATAGCGCACAT	114	NM_001110355.1
	Reverse: TCTCCTCTCTGAGCAAGGCT		
*Actin, beta1 (*β*-actin)*	Forward: CGGACTGTTACCAACACCCA	115	NM_205518.1
	Reverse: TCCTGAGTCAAGCGCCAAAA		

All the experimental groups did not receive any antibiotics.

### Growth Performance

The birds of each replicate were weighed on day 21 of age. Feed intake (FI) was recorded, and feed conversion ratio (FCR) was calculated on day 21 of age on a replication basis.

FI = Feed consumption/number of birds.

FCR = Feed consumption/body weight.

### Sample Collection

On day 21, six birds per treatment (one bird/replicate) were randomly selected (to represent all treatment replicates) and euthanized by cervical dislocation. The current study was designed as a cross-sectional terminal evaluation at the conclusion of the starter and grower phases (21 days). Cecal contents were immediately collected into sterile tubes and stored at −20°C for the microbial count and volatile fatty acid analysis. Ileal samples (approximately 1.5 cm in length of the mid-ileum) were excised and flushed with 0.9% saline to remove all the contents and then fixed in 10% buffered formalin solution for subsequent histomorphological investigations. A section of the mid-ileum (approximately 1.5 cm) was collected, washed with PBS, and immersed in Trizol reagent for subsequent gene expression investigation.

### Histomorphometric Study

Fixed ileal samples were processed, and 4-μm-thick tissue sections were cut out of the paraffin-embedded tissue blocks and stained with hematoxylin and eosin following the protocol of Bancroft and Gamble (28). Stained tissues were examined under a light microscope (Leica DM300 with Leica FLEXACAM C1), whereas representative fields were photographed for morphometrics using Leica LAS X dedicated software. Villus height (VH) and villus width (VW), crypt depth (CD), and muscular thickness were measured. The above-mentioned parameters were measured as the mean of 10 randomly selected parts in each sample. Finally, villus surface area was measured by considering a villus as a cylindrical structure (29) according to the following equation[(2π) × (villus width/2) × (villus height)].

### Bacteriological Examination

One gram of each cecal sample was homogenized in 9 ml of sterilized saline peptone solution and stirred for 30 min to obtain 10^–1^dilution. Decimal serial dilutions were prepared from the previous (10^–1^) to 10^–7^. According to Abd El-Hack et al. (30) and Alagawany et al. (31), an aliquot of 0.1 ml of each dilution was spread over different specific media such as plate count agar (PCA) for total bacterial count (TBC) after incubation at 30°C for 48 h (30). Sabouraud Dextrose Agar (SDA) was used to enumerate total yeast and molds count (TYMC) after incubation at 30°C at 24 h for yeasts and 25°C for 5 days for fungi (32). Violet red bile agar, MacConkey agar, and *Bacillus* cereus agar (Oxoid) were used for total coliforms, *Escherichia coli*, and *B. subtilis*, respectively, after incubation at 37°C for 24 h. *Bacillus* cereus agar (Oxoid) was used for counting *B. subtilis* after incubation for 24 h at 37°C. MRS medium and Chromocult enterococci agar were used for lactic acid bacteria and *Enterococcus* spp., respectively, after incubation at 37°C for 48 h. The microbial counts were converted into log_10_ CFU g^−1^.

### Volatile Fatty Acid Concentration

Immediately following euthanasia, cecal content samples were collected and kept frozen until VFA analysis according to the procedures described by Saad et al. (33). Immediate freezing is a validated protocol in avian studies to stop microbial metabolic activity and prevent short-chain fatty acids from breaking down or evaporating during storage (34). The concentrations of VFA were measured using a mass spectrometer Agilent 5975C, carrier gas helium, column HP-5 ms (30 m × 250 μm × 0.25 μm), and temperature: 35°C/3 min, 5°C/min to 250°C for 3 min, total 49 min, carrier gas helium 1 ml/min constant speed; split ratio 30:1.

### Quantitative Real-Time PCR Analyses

Total RNA was extracted from the ileum using Trizol (Invitrogen; Thermo Fisher Scientific, Inc.) and then reverse-transcribed to cDNA using a High-Capacity cDNA Reverse Transcription Kit (Applied Biosystems™, Waltham, MA, United States) following the manufacturer’s protocol. Real-time RT-PCR was performed in an Mx3005P Real-Time PCR System (Agilent Stratagene, United States) using TOPreal™ qPCR 2 × PreMIX (SYBR Green with low ROX) (Enzynomics, Korea) following the manufacturer’s instructions and according to the previous studies (35–37). The PCR cycling conditions included an initial denaturation at 95°C for 12 min followed by 40 cycles of denaturation at 95°C for 20 s, annealing at 60°C for 30 s, and extension at 72°C for 30 s. All qPCR tests were validated using melting curve analysis, which confirmed a single, distinct peak for each target gene, including β-actin, thereby ensuring high primer specificity and the absence of nonspecific amplification or primer dimers.

### Statistical Analysis

Data were analyzed according to a preplanned experimental set-up using one-way ANOVA (SPSS Inc., 2018).

*Y*_*ij*_ = μ + TRT*_*i*_* + e*_*ij*_*

where *Y*_*ij*_ represents the observation for the dependent variables at the *j*th replicate in the *i*th treatment (*i* = 1 to 10), μ is the overall mean, TRT*_*i*_* is the fixed effect of treatments (*i* = 1 to 10), and e_*ij*_ is the random residual error.

Normality of the distribution was tested with the Shapiro-Wilk normality test, while the homogeneity of variance in the samples was assessed with Levene’s test. The means were compared using Tukey multiple range test. The cage (replicate) served as the experimental unit for comparing growth performance (BW, FI, FCR) (*n* = 6 per treatment), while individuals’ data served as the experimental units for the remaining parameters. One bird was randomly selected per replicate (*n* = 6 per treatment to represent all treatment replicates) to ensure biological diversity. Because only one bird was sampled per cage (replicate), there was no nesting of multiple birds within a single replicate for these specific measurements, which simplifies the variance structure. We acknowledge that the absence of a mixed model for nesting effects represents a simplified variance structure; however, birds were randomly selected from separate replicates to maintain biological independence and minimize potential unstable inference. This approach ensures that the degrees of freedom are correctly partitioned and that Type I error is not inflated by treating individual birds within a cage as independent observations due to the use of Tukey (HSD) for means comparison. Data are presented as means ± SEM, and the two significance was declared at *P* < 0.05.

## Results

### Growth Performance

The effects of *in ovo* inoculation of *B. subtilis*, raffinose, and their combination on the growth performance of broiler chickens at 21 days of age are presented in Table 3. All treated groups had elevated (*p* < 0.01) live body weight (BW) compared to NC. BS1R2, BS1R1, and BS2R2, respectively, and recorded the heaviest weight compared to other groups. *In ovo* supplementation with different levels of *B. subtilis*, raffinose, and their synbiotics improved (*p* < 0.01) FI and FCR during the overall period compared to the control groups.

**TABLE 3:** Effect of *in ovo* inclusion of *Bacillus subtilis*, raffinose, and their synbiotics on growth performance of 21-day-old broilers.

Items	Treatment groups^1^										SEM	*P-value*
	NC	PC	BS1	BS2	R1	R2	BS1R1	BS1R2	BS2R1	BS2R2		
**Body weight, g**												
21 d	909.67^e^	926.75^e^	960.67^cd^	977.00^bc^	946.46^d^	962.47^cd^	990.97^b^	1012.61^a^	977.90^bc^	977.63^bc^	5.60	<0.001
**Feed intake, g**												
0-21 d	1085.99^f^	1090.33^ef^	1116.97^cde^	1123.88^bcd^	1102.40^de^	1124.67^bcd^	1150.23^ab^	1159.36^a^	1127.43^bcd^	1138.06*^abc^*	4.87	<0.001
**Feed conversion ratio**												
0-21 d	1.19^a^	1.18^b^	1.16^cd^	1.15^cde^	1.16^cd^	1.17^bc^	1.16^cd^	1.14^e^	1.15^cde^	1.16^cd^	0.003	<0.001

Means in a row with different superscripts are significantly different (*p* < 0.05).

^1^NC, non-injected group; PC, sterile distilled water; BS1 = *Bacillus subtilis* 4 × 10^5^/egg; BS2 = *Bacillus subtilis* 4 × 10^6^/egg; R1 = Raffinose 2 mg/egg; R2 = Raffinose 3 mg/egg; BS1R1 = (*Bacillus subtilis* 4 × 10^5^ + Raffinose 2 mg)/egg; BS1R2 = (*Bacillus subtilis* 4 × 10^5^ + Raffinose 3 mg)/egg; BS2R1 = (*Bacillus subtilis* 4 × 10^6^ + Raffinose 2 mg)/egg; BS2R2 = (*Bacillus subtilis* 4 × 10^6^ + Raffinose 3 mg)/egg. SEM, standard error of means.

### Cecal Volatile Fatty Acid Concentration

Concentrations of major VFA in cecal contents of 21-day-old broilers as influenced by *in ovo* treatments are presented in Table 4. Concentrations of butyric, pentanoic, propionic, and isobutyric acids were elevated (*p* < 0.001) in R1, R2, BS2R1, and BS2R2 groups. In contrast, levels of isovaleric and acetic acids were increased (*p* < 0.001) only in R1 and BS2R1 groups compared to the control groups. R1 group recorded the highest cecal VFA levels, followed by BS2R1.

**TABLE 4:** Effect of *in ovo* inclusion of *Bacillus subtilis*, raffinose, and their synbiotics on cecal volatile fatty acid concentrations at 21-day-old broilers.

Items (μ mol/g)	Treatment groups^1^										SEM	*P-*value
	NC	PC	BS1	BS2	R1	R2	BS1R1	BS1R2	BS2R1	BS2R2		
Butyric acid	1.85^d^	1.85^d^	1.87^d^	1.86^d^	2.74^a^	2.04^c^	1.90^d^	1.89^d^	2.46^b^	2.03^c^	0.05	<0.001
Isovaleric acid	0.99^b^	1.02^b^	0.97^b^	0.95^b^	1.80^a^	0.80^b^	0.99^b^	0.97^b^	1.51^a^	1.10^b^	0.06	<0.001
Pentanoic acid	0.40^d^	0.42^d^	0.41^d^	0.40^d^	1.22^a^	0.54^c^	0.41^d^	0.41^d^	0.91^b^	0.53^c^	0.05	<0.001
Acetic acid	32.23^c^	32.16^c^	30.43^d^	32.20^c^	33.64^a^	32.52^c^	30.43^d^	32.28^c^	33.10^b^	32.41^c^	0.18	<0.001
Propionic acid	1.52^d^	1.53^d^	1.48^d^	1.48^d^	2.65^a^	1.72^c^	1.51^d^	1.52^d^	2.23^b^	1.67^c^	0.07	<0.001
Isobutyric acid	0.92^d^	0.93^d^	0.92^d^	0.94^d^	1.96^a^	1.10^c^	0.92^d^	0.92^d^	1.565^b^	1.07^c^	0.06	<0.001

Means in a row with different superscripts are significantly different (*p* < 0.05).

^1^NC, non-injected group; PC, sterile distilled water; BS1, *Bacillus subtilis* 4 × 10^5^/egg; BS2 = *Bacillus subtilis* 4 × 10^6^/egg; R1 = Raffinose 2 mg/egg; R2 = Raffinose 3 mg/egg; BS1R1 = (*Bacillus subtilis* 4 × 10^5^ + Raffinose 2 mg)/egg; BS1R2 = (*Bacillus subtilis* 4 × 10^5^ + Raffinose 3 mg)/egg; BS2R1 = (*Bacillus subtilis* 4 × 10^6^ + Raffinose 2 mg)/egg; BS2R2 = (*Bacillus subtilis* 4 × 10^6^ + Raffinose 3 mg)/egg. SEM, standard error of means.

### Microbial Enumeration

Cecal microbial enumeration of broilers at 21 days of age was remarkably influenced (*p* < 0.001) by *in ovo* administration of *B. subtilis*, raffinose, and their synbiotics (Table 5). Cecal population of *B. subtilis* and lactic acid bacteria was elevated (*p* < 0.001) by *in ovo* probiotics and synbiotics compared to NC. The count of total molds and yeast was not altered among experimental groups. The population of *E. coli, Enterococcus* spp., total coliform, and the total bacterial count were decreased (*p* < 0.001) in the cecal contents of all treated birds compared to the control groups.

**TABLE 5:** Effect of *in ovo* inclusion of *Bacillus subtilis*, raffinose, and their synbiotics on cecal microbial population of 21-day-old broilers.

Item (log^10^CFU/g)	Treatment groups^1^										SEM	*P-value*
	NC	PC	BS1	BS2	R1	R2	BS1R1	BS1R2	BS2R1	BS2R2		
Total bacterial count	10.19^a^	10.08^b^	9.65^e^	9.55^f^	9.92^c^	9.76^d^	9.49^fg^	9.56^ef^	9.29^h^	9.42^g^	0.05	< 0.001
Total yeasts and molds count	5.07	4.93	4.31	4.15	4.39	4.33	4.65	4.83	4.42	4.52	0.16	0.137
Lactic acid bacteria	5.37^de^	5.26^e^	6.01^c^	6.75^a^	5.38^de^	5.61^d^	6.33^b^	6.24^bc^	6.52^ab^	6.46^ab^	0.10	< 0.001
*B. subtilis*	5.75^d^	5.66^d^	7.82^a^	7.86^a^	5.92^d^	5.87^d^	7.51^ab^	7.10^bc^	7.71^a^	6.95^c^	0.17	< 0.001
Total coliform	6.94^a^	6.89^b^	6.53^g^	6.38^h^	6.62^fg^	6.56^g^	6.71^d^	6.78^c^	6.59^gh^	6.65^e^	0.03	< 0.001
*E.coli*	5.63^a^	5.57^a^	4.67^g^	4.47^h^	5.06^e^	4.86^f^	5.35^c^	5.45^b^	5.16^d^	5.22^d^	0.07	< 0.001
*Enterococcus spp.*	6.19^a^	5.95^b^	4.92^h^	4.81^i^	5.22^g^	5.35^f^	5.75^d^	5.85^c^	5.46^e^	5.69^d^	0.08	< 0.001

Means in a row with different superscripts are significantly different (*p* < 0.05).

^1^NC, non-injected group; PC, sterile distilled water; BS1 = *Bacillus subtilis* 4 × 10^5^/egg; BS2 = *Bacillus subtilis* 4 × 10^6^/egg; R1 = Raffinose 2 mg/egg; R2 = Raffinose 3 mg/egg; BS1R1 = (*Bacillus subtilis* 4 × 10^5^ + Raffinose 2 mg)/egg; BS1R2 = (*Bacillus subtilis* 4 × 10^5^ + Raffinose 3 mg)/egg; BS2R1 = (*Bacillus subtilis* 4 × 10^6^ + Raffinose 2 mg)/egg; BS2R2 = (*Bacillus subtilis* 4 × 10^6^ + Raffinose 3 mg)/egg. SEM, standard error of means.

### Ileal Histomorphometry

**FIGURE 1:**
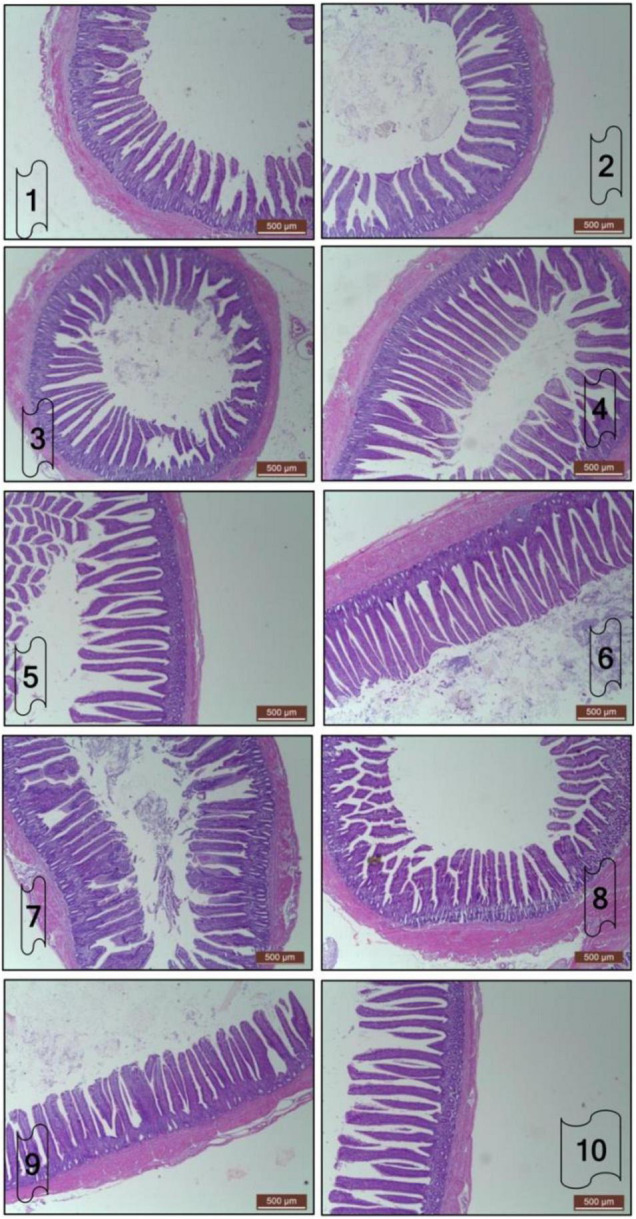
Effect of *in ovo* inclusion of *Bacillus subtilis*, raffinose, and their synbiotics on ileal histomorphometry of 21-day-old broilers. Each number on the figure means the following groups: 1 = non-injected group; 2 = sterile distilled water; 3 = *Bacillus subtilis* 4 × 10^5^/egg; 4 = *Bacillus subtilis* 4 × 10^6^/egg; 5 = Raffinose 2 mg/egg; 6 = Raffinose 3 mg/egg; 7 = (*Bacillus subtilis* 4 × 10^5^ + Raffinose 2 mg)/egg; 8 = (*Bacillus subtilis* 4 × 10^5^ + Raffinose 3 mg)/egg; 9 = (*Bacillus subtilis* 4 × 10^6^ + Raffinose 2 mg)/egg; 10 = (*Bacillus subtilis* 4 × 10^6^ + Raffinose 3 mg)/egg. Images were captured with light microscopy. Scale bar indicates 500 μm.

The architecture of ileal samples of 21-day-old broilers *in ovo* treated with *B. subtilis*, raffinose, and their synbiotics is presented in Figure 1 and Table 6. Ileal VH was significantly increased (*p* < 0.001) in BS1, R2, and BS2R2 compared to NC. The highest value of ileal VH has observed in the group that received a high prebiotic level. However, VW and CD were not significantly affected by the *in ovo* supplements. Significant differences (*p* < 0.05) were found in the values of muscular thickness among the different groups. BS2R2 group had the highest value compared to the other groups. Values of villus surface area showed an increasing trend compared to the control groups. However, this increase was not statistically significant (*p* > 0.05).

**TABLE 6:** Effect of *in ovo* inclusion of *Bacillus subtilis*, raffinose, and their synbiotics on ileal morphology of 21-day-old broilers.

Items (μm)	Treatment groups^1^										SEM	*P-*value
	NC	PC	BS1	BS2	R1	R2	BS1R1	BS1R2	BS2R1	BS2R2		
Villus height	717.48^cd^	522.61^e^	888.82^ab^	760.30^bc^	710.35^cd^	915.98^a^	607.11^de^	700.38^cd^	661.33^cde^	896.25^ab^	27.16	< 0.001
Villus width	92.48	119.91	101.38	86.77	128.68	101.37	125.07	118	123.86	121.84	4.222	0.239
Crypt depth	87.03	65.58	85.8	102.7	69.73	93.81	117.33	81.86	91.5	120.4	4.713	0.121
Muscularis thickness	203.37^b^	123.05^c^	172.89^bc^	182.31^bc^	126.81^c^	142.48^c^	181.03^bc^	201.74^b^	220.17^ab^	280.73^a^	11.23	0.029
Surface area (μm^2^)	20705.00	196670.00	285050.00	207310.00	290910.00	290210.00	235530.00	260080.00	257290.00	343040.00	32240.00	0.083

Means in a row with different superscripts are significantly different (*p* < 0.05).

^1^NC, non-injected group; PC, sterile distilled water; BS1 = *Bacillus subtilis* 4 × 10^5^/egg; BS2 = *Bacillus subtilis* 4 × 10^6^/egg; R1, Raffinose 2 mg/egg; R2, Raffinose 3 mg/egg; BS1R1 = (*Bacillus subtilis* 4 × 10^5^ + Raffinose 2 mg)/egg; BS1R2 = (*Bacillus subtilis* 4 × 10^5^ + Raffinose 3 mg)/egg; BS2R1 = (*Bacillus subtilis* 4 × 10^6^ + Raffinose 2 mg)/egg; BS2R2 = (*Bacillus subtilis* 4 × 10^6^ + Raffinose 3 mg)/egg. SEM, standard error of means.

### Ileal Gene Expression

#### Relative Expression of Intestinal Function-Related Genes

**FIGURE 2:**
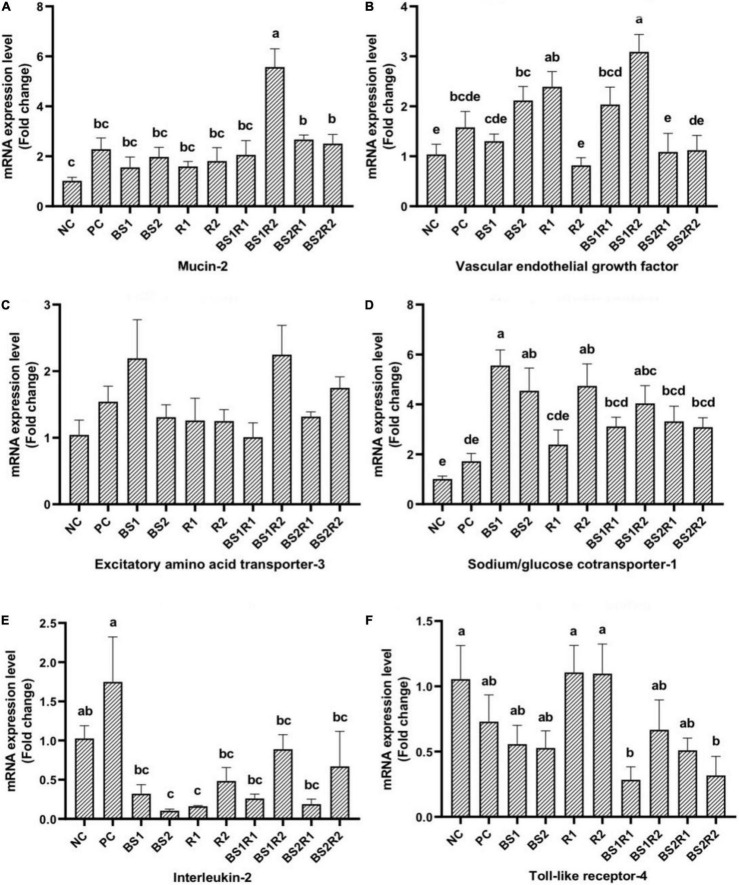
Effect of *in ovo* inclusion of *Bacillus subtilis*, raffinose, and their synbiotics on ileal gene expression of 21-day-old broilers. **(A,B)** Intestinal function-related, **(C,D)** nutrient transporter genes, and **(E,F)** immune-related genes. NC, non-injected group; PC, sterile distilled water; BS1 = *Bacillus subtilis* 4 × 10^5^/egg; BS2 = *Bacillus subtilis* 4 × 10^6^/egg; R1 = Raffinose 2 mg/egg; R2 = Raffinose 3 mg/egg; BS1R1 = (*Bacillus subtilis* 4 × 10^5^ + Raffinose 2 mg)/egg; BS1R2 = (*Bacillus subtilis* 4 × 10^5^ + Raffinose 3 mg)/egg; BS2R1 = (*Bacillus subtilis* 4 × 10^6^ + Raffinose 2 mg)/egg; BS2R2 = (*Bacillus subtilis* 4 × 10^6^ + Raffinose 3 mg)/egg. Data are presented as the mean values with their standard errors. Gene expression differences were evaluated using fold changes compared to the non-injected group. The significance was declared at *p* < 0.05. Bars with different letters represent significant differences among the different groups.

The mRNA expression of mucin-2 and vascular endothelial growth factor genes in the ileum of broilers in different experimental groups is illustrated in Figures 2A,B. The mRNA expression of mucin-2 was elevated significantly (*p* < 0.05) in BS1R2, BS2R1, and BS2R2, and numerically in the rest of the groups compared to NC. Vascular endothelial growth factor (VEGF) expression was increased (*p* < 0.05) in BS2, R1, BS1R1, and BS1R2 compared to NC. BS1R2 recorded the highest mRNA expression of both genes.

#### Relative Expression of Nutrient Transporter Genes

The mRNA expression of nutrients transportation-related genes (EAAT-3 and SGLT-1) in the ileum of broilers *in ovo* treated with *B. subtilis*, raffinose, and their synbiotics is shown in Figures 2C,D. The gene expression of EAAT-3 was not affected by *in ovo* supplements with a numerical increase in BS1 and BS1R2 groups compared to the control groups. SGLT-1 expression was upregulated (*p* < 0.05) in all treated birds except those of the R1 group compared to NC.

#### Relative Expression of Immune-Related Genes

The ileal mRNA expression of immune-related genes (interleukin (IL)-2 and toll-like receptor (TLR)-4) of broilers *in ovo* inoculated with *B. subtilis*, raffinose, and their combination is depicted in Figures 2E,F. The mRNA expressions of IL-2 and TLR-4 were downregulated (*p* < 0.05) in BS2 and R1 for IL-2 and BS1R1 and BS2R2 for TLR-4 and decreased numerically in the remaining groups compared to the control groups.

## Discussion

Early colonization by beneficial bacteria or the inclusion of vital nutrients that encourage the growth of these beneficial microorganisms may play a major role in improving growth performance and reducing the occurrence of infections in chickens fed with no antibiotic growth promoters and enhancing the sustainability of broiler production (39). In a research review, Shehata et al. (7) discussed how *in ovo* administration of probiotics and prebiotics alters the growth performance, gut microbiota, and gut health of broiler chickens (7). In the present study, *in ovo* inoculation of *B. subtilis*, raffinose, and their combination improved BW, FI, and FCR compared to the untreated group. The improvement effects of these supplements could be due to their role in enhancing the health status of the intestine *via* increasing the population of beneficial bacteria, decreasing the population of harmful bacteria, regulating the expression of several ileal genes, and improving intestinal morphology (22, 26). *B. subtilis* is spore-forming bacteria that can tolerate harsh environmental conditions, colonize birds’ gut, and increase the activities of exogenous digestive enzymes, including lipase, protease, and amylase, which contribute to increasing nutrients digestion and absorption (18, 40). Tavaniello et al. (41) revealed that the positive impact of raffinose-injected *in ovo* on growth performance might be attributed to their ability to stimulate the early development of intestinal microbiota *via* increasing the population of beneficial bacteria such as lactobacilli and bifidobacteria and preventing colonization of pathogens. The proteome and cellular adhesion analyses showed improved adhesive properties of the beneficial bacteria (*Lactobacillus acidophilus*) grown on raffinose. This improvement was positively associated with differential protein abundance (elongation factor G and myosin cross-reactive antigen), indicating the positive interaction with mucin and host intestinal epithelial cells (42). Moreover, a previous study confirmed the prebiotic properties of raffinose family oligosaccharides *via* promoting the growth of *Bifidobacterium bifidum* and *L. acidophilus* (43). Additionally, administration of *B. subtilis*, prebiotics, or synbiotics caused an increase in the activity of pancreatic enzymes and thyroid hormones, which enhance the utilization of nutrients to maintain optimum growth performance of treated chickens (18, 44). Earlier investigations have demonstrated that *Bacillus* spp.-based probiotic is resistant to intestinal biochemical conditions *in vitro* and *in vivo* in chickens (45, 46). The ability of *Bacillus*-based probiotic to improve gut morphology and the gut microbiota structure may effectively contribute to increasing nutrient utilization and enhancing the immune responses, leading to improved growth performance. A previous study showed that *Bacillus* spp.-based probiotic produces various enzymes and antibacterial substances, which can enhance growth performance by improving nutrient digestibility, modulating gut viscosity, and enhancing intestinal integrity (47). In conformation with our results, Sen et al. (48) documented that BWG and FCR were increased linearly by increasing the dietary level of *B. subtilis*. Abdel-Moneim et al. (18) and Jeong and Kim (49) reported that the inclusion of *B. subtilis* spores improved weight gain of broilers. Additionally, Tavaniello et al. (41) and Bednarczyk et al. (50) reported that *in ovo* inoculation of raffinose improved the growth performance of Ross-308 chickens. Furthermore, chickens *in ovo* injected with prebiotics and synbiotics recorded heavier weights than the control groups (21). On the contrary, other studies reported non-significant effects of *in ovo* supplementation with *B. subtilis* and raffinose on the growth performance of broiler chickens (12, 16, 26, 51). These inconsistencies among studies might be attributed to various factors, including dosage of bioactive substances, sources, viability, administration route, environmental stressors, or sample size (12, 21). Intra-amniotic administration of *B. subtilis* fermentation extract (10 × 10^6^ CFU) on day 18.5 of incubation did not affect the growth performance of Cobb 500 broiler chickens (12). Likewise, *B. subtilis* (10^7^ CFU) injected on day 18 of incubation into the amnion had no significant effect on the growth performance of Cobb 500 broilers (16). In a previous study, inoculation of raffinose (1.5, 3.0, and 4.5 mg/egg) showed a numerical improvement in growth performance of broilers without any significant differences between the different groups (26). *In ovo* injection of 1.9 mg/egg raffinose (extracted from the seeds of *Lupinus luteus* L) did not affect the growth performance of broilers on the slaughter age (51).

The short-chain fatty acid (SCFA) and VFA are the by-products of cecal microbial fermentation and play vital roles in reducing gut pathogens, the functionality of the intestinal tract, and birds’ energy metabolism (52). In the present study, *in ovo* supplements elevated the levels of butyric, pentanoic, propionic, isobutyric, isovaleric, and acetic acids. These results are in agreement with those obtained recently by Oladokun et al. (12), who reported, for the first time, the effect of *in ovo*-delivered *B. subtilis* on concentrations of SCFA in broilers’ cecum. The authors found a consistent increase in VFA concentrations due to *in ovo* probiotics treatment. Moreover, dietary supplementation of soybean fermented with *B. subtilis var. natto* increased acetic acid and the total VFA concentrations (53). Similarly, administration of *L. agilis* and *L. salivarius* ssp. *salicinius* increased butyrate and propionate concentrations (52). Adding *B. subtilis* or *B. licheniformis* to chicken feed enhanced SCFA production *via* altering the microbiota structure in the chicken’s gut (54). SCFAs, in addition to being the primary source of energy for colonocytes, play a crucial role in maintaining health and modulating immunological and inflammatory responses (55). Nevertheless, further studies are needed to fully understand the effect of *in ovo* inoculation of probiotics and prebiotics due to the limited studies investigating this point (12).

The antimicrobial effects of *B. subtilis*, raffinose, and their synbiotics are well-presented in the current study. The inhibitory activity of these supplements is attributed to their ability to produce antibacterial and antifungal substances, including bacteriocins, bacteriocin-like substances, acetic acid, hydrogen peroxide, carbon dioxide, diacetyl, and lactic acid (7, 56). Intestinal immunity modulation (7), fat storage regulation (57), dietary fiber utilization (58), and competitive insularity of pathogenic bacteria (59) are other functional properties of the antimicrobial role of the *in ovo* supplements. The impacts of probiotics and prebiotics on modifying the intestinal microbiota composition by suppressing pathogen numbers and elevating counts of beneficial microorganisms were previously documented (14). Oh et al. (60) and Li et al. (8) reported the potential of *B. subtilis* to improve the richness of bacterial species, particularly from phylum Bacteroidetes. Our results are in agreement with those of Abdel-Moneim et al. (23), who noticed a reduction in total coliform and total bacterial counts and increased counts of bifidobacteria and lactic acid bacteria in ileal contents due to the *in ovo* injection of four bifidobacteria strains. Dietary supplementation of *B. toyonensis* and *Bifidobacterium bifidum* depressed the population of intestinal coliform, *E. coli*, and total bacterial count. Serafini et al. (61) also reported the inhibitory activity of probiotics on pathogenic bacteria such as *E. coli* and *Cronobacter sakazakii*. Furthermore, Pacifici et al. (62) and Stadnicka et al. (51) reported that *in ovo* administration of raffinose reduced pathogenic bacteria like Clostridia and *E. coli* and increased the population of beneficial bacteria like bifidobacteria and lactobacilli. Davani-Davari et al. (63) stated in their recent review on prebiotic that more study is needed to understand the impact of raffinose on gut flora fully. The crucial role of raffinose in enhancing the fermentation by *Lactobacillus* and *Bifidobacteria* and the production of SCFAs improves the competition with the pathogens. It improves the intestinal histomorphology and immune-related genes. This has been demonstrated before; for instance, treatment with beneficial bacteria reduced the virulence of pathogenic bacteria by promoting the formation of SCFAs in the gut (64). We propose that a similar mode of action may explain the results given here, with the resulting improvement in broiler chicken health and performance. However, the administration of Amprolium (75 g/100 L) during the second week was implemented as a standard commercial prophylactic measure to manage coccidial risk (65). While anticoccidials can influence gut microbiota and immune responses, Amprolium was applied uniformly across all experimental groups, thereby balancing its physiological effects within the randomized design. Furthermore, current research suggests that *B. subtilis* and Amprolium are not mutually exclusive; *B. subtilis* does not rely on the thiamine-dependent pathways targeted by Amprolium in *Eimeria* parasites (65, 66). Therefore, the use of Amprolium in this study reflects a realistic commercial environment and further validates the effectiveness of the synbiotic under standard production stressors, with no significant confounding interactions. However, we suggest that further investigation is required to improve combination therapies in clinical settings.

The gastrointestinal tract is the main interface between the birds and their nutritional environment, which allows it to play a pivotal role in the development and growth of hatchlings. Improving the intestinal microarchitecture of broiler chicks enhances feed utilization and nutrient uptake. The roles of probiotics and prebiotics in improving gut health and structure have been well-documented in previous studies (9, 51, 67–69). In the present study, ileal VH was improved in broilers *in ovo* treated with *B. subtilis*, raffinose, and their synbiotic. These results support the previous study of Berrocoso et al. (26) who noticed an improvement in the ileum mucosa development by *in ovo* injection of raffinose in the air sac. The same observations after *in ovo* inoculation of raffinose were reported in a recent study (51). Furthermore, Elbaz et al. (9), Abdel-Moneim et al. (23), and Abou-Kassem et al. (70) reported that administration of probiotics strains increased VH and reduced CD regardless of the route of administration. According to Abdel-Moneim et al. (18), the elevation in VH is accompanied by a tendency to reduce CD, higher activity of the digestive enzymes, and better absorption.

Additionally, longer lifespan and faster healing of enterocytes are associated with longer villi and shallower crypts, contributing to better nutrient utilization and improved growth performance (71, 72). Commensal bacteria can ferment raffinose, and the generated VFAs lower the pH of the intestines. Butyrate, one of the SCFAs produced during this fermentation, promotes the development of intestinal epithelial cells, which enhances nutrient uptake (73).

Maintaining gut integrity and mucin secretion is crucial to reducing pathogenic invasion and improving nutrient digestion and absorption. Mucins are mainly glycoproteins that lubricate and protect the intestinal epithelial surface from pathogens’ adhesion and invasion. Mucin production is encoded by the mucin-2 gene, which plays an important immunological role and is mediated by Th2 cytokines and T lymphocytes (74). Mucin dynamics are greatly influenced by intestinal microbiota and probiotic bacteria (7, 75). Mucin biosynthesis, turnover, and secretion are affected by intestinal microbiota and their metabolites, as they stimulate mucin gene expression *via* prostaglandin production (76). *VEGF* gene is essential in regulating tissue capillarity, improving their endogenous regeneration and vascularity, and lowering tissue fibrosis (77). The better expression of *VEGF* gene enhances blood supply to the intestine, leading to better nutrient absorption. Our results revealed upregulation in the expression of *mucin-2* and *VEGF* genes in *in ovo*-treated birds. In line with our findings, Pender et al. (78) demonstrated that *in ovo* supplementation of probiotics elevates the expression of mucin-2 at 22 days post-hatch. Similarly, Majidi-Mosleh et al. (16) revealed that *in ovo* inoculation of *B. subtilis* into the amniotic fluid upregulated the intestinal mucin-2 gene expression in broilers at 3 days of age. Tsirtsikos et al. (79) reported a linear increase in mucus layer thickness with the addition of probiotics on day 12 and 42 of age.

Brush-border membrane genes are functional genes found on the brush border of enterocytes and used as biomarkers of intestinal capability for digestion and absorption and general tissue functionality (80, 81). SGLT1 is a glucose transporter, whereas EAAT3 is a glutamate and aspartate transporter, and both are located in the intestinal enterocytes. Prior to hatch, because of the low functional gene expression of brush-border membrane genes, such as *SGLT1* and *EAAT3*, chicken embryos have a low ability to digest and absorb nutrients (82). Yalcin et al. (83) reported the upregulation of SGLT1 and PepT1 expression after hatch in the first 30 h. Therefore, *in ovo* and early nutrition are crucial for the intestinal development. Our results revealed non-significant alteration in EAAT3 expression, whereas SGLT1 expression was elevated in all treated birds. These results agreed with those of Pacifici et al. (62), who reported that intra-amniotic administration of raffinose upregulated SGLT1 expression significantly. Similarly, dietary supplementation of multi-strain probiotics upregulated the expression of SGLT1 in broilers at 14 days post-hatch (84). The upregulated expression of brush-border membrane functional genes indicates the higher function and development of the small intestine and better nutrient absorption, leading to improving the growth performance of birds.

Early establishment of beneficial microbiota and modulation of the immune system in poultry by *in ovo* administration of probiotics, prebiotics, or synbiotics contribute to increasing general health, well-being, and performance of birds. These supplements also contribute to eliminating the use of prophylactic drugs. The immunomodulating capabilities of these supplements are represented in several reports (9, 78, 84). TLR-2 is a pathogen recognition receptor expressed on infectious agents to recognize the molecular patterns associated with microbes and play a vital role in initiating and regulating the innate immune response. IL-2 plays a fundamental role in stimulating the proliferation of T and B lymphocytes and natural killer cells as a pro-inflammatory cytokine. In this study, we evaluated the expression of TLR-2 and IL-2 as indicators of the effect of *in ovo* supplements on innate and adaptive immune responses, respectively. Both TLR-4 and IL-2 were downregulated in the present study by *in ovo*-delivered supplements. The downregulation of the expression of these genes could be a result of the inhibitory impacts of *in ovo* supplements to pathogen colonization, which eliminates the need for TLR-4 and IL-2 induction. Correlating with our findings, Pender et al. (78) reported that *in ovo* injection of probiotics mixture of Lactobacilli, Enterococci, and Bifidobacteria strains suppressed the intestinal expression of TLR-2, TLR-4, IL-4, and IL-13 in broilers. Berrocoso et al. (26) revealed that *in ovo* injection of raffinose upregulated the expression of chB6 and CD3 in the gut of broiler chickens, whereas expressions of TLR-4, IL-1β, and IL-10 were not altered. The down-regulated expression of innate immunity-related genes might be attributed to the absence of pathogenic infections in treated groups. The authors attributed this lack of improvement of innate immunity to the absence of pathogenic infections because the birds were reared in a sanitary environment. We selected β-actin as the reference gene for normalizing the expression of intestinal function (Mucin-2, VEGF), nutrient transporter (SGLT1, EAAT3), and immune (IL-2, TLR-4) genes due to its demonstrated stability in chicken ileal studies and its prevalent use among leading experts in the field (85, 86). Nonetheless, we acknowledge that using a single housekeeping gene without rigorous validation may not adequately satisfy MIQE reporting guidelines. We have used the standard 2^−ΔΔCT^ method for relative quantification; however, in future investigations, we intend to incorporate additional reference genes to enhance the robustness and transparency of our gene expression results.

## Conclusion

*In ovo* inoculation of different levels of *B. subtilis* (4 × 10^5^ and 4 × 10^6^/egg), raffinose (2 and 3 mg), and their combinations on 300 h of incubation can improve the growth performance, intestinal morphology, cecal microbial population, and cecal VFA of broiler chickens, enhancing the sustainability of broiler production up to 21 days. *In ovo* supplementation plays an immunomodulatory role *via* modulating the expression of some ileal immune-related genes. Furthermore, our data showed that *in ovo* supplementation of *B. subtilis*, raffinose, and their combinations can upregulate the mRNA expression of intestinal function-related genes and nutrient transporter-related genes. In conclusion, *in ovo* supplementation with the *B. subtilis*, raffinose, or their combinations provided beneficial changes to growth performance and gut health of broiler chickens.

## Data Availability Statement

The original contributions presented in the study are included in the article/Supplementary material, further inquiries can be directed to the corresponding authors.

## Ethics Statement

The animal study was reviewed and approved by the Central Animal Ethical Committee of Banaras Hindu University, Varanasi, India.

## Author Contributions

ASh and VP designed the experiment. ASh conducted the animal experiment. ASh, TK, MAA, ES, ME-S, and ASa conducted the detection and analysis works. ASh and A-MA-M prepared the manuscript with input obtained from YA, AA, MMA, and RE. All authors participated in the discussion and editing of the manuscript.

## Conflict of Interest

The authors declare that the research was conducted in the absence of any commercial or financial relationships that could be construed as a potential conflict of interest.

## Publisher’s Note

All claims expressed in this article are solely those of the authors and do not necessarily represent those of their affiliated organizations, or those of the publisher, the editors and the reviewers. Any product that may be evaluated in this article, or claim that may be made by its manufacturer, is not guaranteed or endorsed by the publisher.
