# Correction: *In ovo* inoculation of *Bacillus subtilis* and raffinose affects growth performance, cecal microbiota, volatile fatty acid, ileal morphology and gene expression, and sustainability of broiler chickens (*Gallus gallus*)

**DOI:** 10.3389/fnut.2026.1867465

**Published:** 2026-06-12

**Authors:** Abdelrazeq M. Shehata, Vinod K. Paswan, Youssef A. Attia, Mohammed Sh. Abougabal, Tarek Khamis, Amany I. Alqosaibi, Mashael M. Alnamshan, Reda Elmazoudy, Mohamed A. Abaza, Ehab A. A. Salama, Mohamed T. El-Saadony, Ahmed M. Saad, Abdel-Moneim Eid Abdel-Moneim

**Affiliations:** 1 Department of Animal Production, Faculty of Agriculture, Al-Azhar University, Cairo, Egypt; 2 Department of Dairy Science and Food Technology, Institute of Agricultural Sciences, Banaras Hindu University, Varanasi, India; 3 Department of Animal and Poultry Production, Faculty of Agriculture, Damanhour University, Damanhour, Egypt; 4 Sustainable Agriculture Research Group, Department of Agriculture, Faculty of Environmental Sciences, King Abdulaziz University, Jeddah, Saudi Arabia; 5 Department of Pharmacology, Faculty of Veterinary Medicine, Zagazig University, Zagazig, Egypt; 6 Laboratory of Biotechnology, Faculty of Veterinary Medicine, Zagazig University, Zagazig, Egypt; 7 Biology Department, College of Science, Imam Abdulrahman Bin Faisal University, Dammam, Saudi Arabia; 8 Avian and Rabbit Diseases Department, Faculty of Veterinary Medicine, Benha University, Banha, Egypt; 9 Agricultural Botany Department, Faculty of Agriculture (Saba Basha), Alexandria University, Alexandria, Egypt; 10 Department of Agricultural Microbiology, Faculty of Agriculture, Zagazig University, Zagazig, Egypt; 11 Biochemistry Department, Faculty of Agriculture, Zagazig University, Zagazig, Egypt; 12 Biological Applications Department, Nuclear Research Center, Egyptian Atomic Energy Authority, Abu Zaabal, Egypt

**Keywords:** bioactive compounds, *in ovo* feeding, gut microbiota, volatile fatty acid, ileal architecture, gene expression, sustainability, broiler chickens

In the published article, the **Data availability statement** was erroneously given as “The datasets presented in this study can be found in online repositories. The names of the repository/repositories and accession number(s) can be found in the article/supplementary material”. The correct **Data availability statement** is as follows: “The original contributions presented in the study are included in the article/**Supplementary material**, further inquiries can be directed to the corresponding authors.”

In the published article, “Wagner et al., 2017” was mistakenly omitted from the reference list: “Wagner AO, Markt R, Puempel T, Illmer P, Insam H, Ebner C. Sample preparation, preservation, and storage for volatile fatty acid quantification in biogas plants. *Eng Life Sci*. (2017) 17:132–9. doi: 10.1002/elsc.201600095”

The reference list has been updated and the citation for “Wagner et al., 2017” (34) has been inserted in **Materials and methods**, *Volatile fatty acid concentration*, Paragraph 1, which has been revised as follows:

“Immediately following euthanasia, cecal content samples were collected and kept frozen until VFA analysis according to the procedures described by Saad et al. (33). Immediate freezing is a validated protocol in avian studies to stop microbial metabolic activity and prevent short-chain fatty acids from breaking down or evaporating during storage (34). The concentrations of VFA were measured using a mass spectrometer Agilent 5975C, carrier gas helium, column HP-5 ms (30 m × 250 μm × 0.25 μm), and temperature: 35 °C/3 min, 5 °C/min to 250 °C for 3min, total 49 min, carrier gas helium 1 ml/min constant speed; split ratio 30:1.”

In the published article, “Chalalai et al., 2025” was mistakenly omitted from the reference list: “Chalalai T, Promsut W, Hinkhao K, Hengphrathani T, Sangsakul K, Bhavabhutanon N, et al. Effects of probiotics and amprolium on performance, lesion scores, oocyst shedding, and histopathological changes in *Eimeria tenella*-infected broiler chickens. *Vet World*. (2025) 18:1400–10. doi: 10.14202/vetworld.2025.1400-1410”

Likewise, “Sudan et al., 2021” was mistakenly omitted from the reference list: “Sudan S, Flick R, Nong L, Li J. Potential probiotic *Bacillus subtilis* isolated from a novel niche exhibits broad range antibacterial activity and causes virulence and metabolic dysregulation in enterotoxic *E. coli*. *Microorganisms*. (2021) 9:1483. doi: 10.3390/microorganisms9071483”

The reference list has been updated and “Chalalai et al., 2025” (65) and “Sudan et al., 2021” (66) have been cited in a new passage at the end of **Discussion**, Paragraph 3:

“We propose that a similar mode of action may explain the results given here, with the resulting improvement in broiler chicken health and performance. However, the administration of Amprolium (75 g/100 L) during the second week was implemented as a standard commercial prophylactic measure to manage coccidial risk (65). While anticoccidials can influence gut microbiota and immune responses, amprolium was applied uniformly across all experimental groups, thereby balancing its physiological effects within the randomized design. Furthermore, current research suggests that *B. subtilis* and amprolium are not mutually exclusive; *B. subtilis* does not rely on the thiamine-dependent pathways targeted by amprolium in *Eimeria* parasites (65, 66). Therefore, the use of amprolium in this study reflects a realistic commercial environment and further validates the effectiveness of the synbiotic under standard production stressors, with no significant confounding interactions. However, we suggest that further investigation is required to improve combination therapies in clinical settings.”

In the published article, “De Boever et al., 2008” was mistakenly omitted from the reference list: “De Boever S, Vangestel C, De Backer P, Croubels S, Sys SU. Identification and validation of housekeeping genes as internal control for gene expression in an intravenous LPS inflammation model in chickens. *Vet Immunol Immunopathol*. (2008) 122:312–7. doi: 10.1016/j.vetimm.2007.12.002”

“Habashy et al., 2017” was also mistakenly omitted from the reference list: “Habashy WS, Milfort MC, Adomako K, Attia YA, Rekaya R, Aggrey SE. Effect of heat stress on amino acid digestibility and transporters in meat-type chickens. *Poult Sci*. (2017) 96:2312–9. doi: 10.3382/ps/pex027”

The reference list has been updated and “De Boever et al., 2008” (85) and “Habashy et al., 2017” (86) have been cited in a new passage at the end of **Discussion**, Paragraph 8:

“The authors attributed this lack of improvement of innate immunity to the absence of pathogenic infections because the birds were reared in a sanitary environment. We selected -actin as the reference gene for normalizing the expression of intestinal function (Mucin-2, VEGF), nutrient transporter (SGLT1, EAAT3), and immune (IL-2, TLR-4) genes due to its demonstrated stability in chicken ileal studies and its prevalent use among leading experts in the field (85, 86). Nonetheless, we acknowledge that using a single housekeeping gene without rigorous validation may not adequately satisfy MIQE reporting guidelines. We have used the standard 2^−Δ*ΔCT*^ method for relative quantification; however, in future investigations, we intend to incorporate additional reference genes to enhance the robustness and transparency of our gene expression results.”

There was a mistake in Table 5 as published. In row 4 of Table 5, “*B. subtills*” was used instead of “*B. subtilis”* The corrected table appears below.

**Table 5:** Effect of *in ovo* inclusion of *Bacillus subtilis*, raffinose, and their synbiotics on cecal microbial population of 21-day old broilers.

Item (log^10^CFU/g)	Treatment groups^1^										SEM	*P*-value
	NC	PC	BS1	BS2	R1	R2	BS1R1	BS1R2	BS2R1	BS2R2		
Total bacterial count	10.19^a^	10.08^b^	9.65^e^	9.55^f^	9.92^c^	9.76^d^	9.49^fg^	9.56^ef^	9.29^h^	9.42^g^	0.05	< 0.001
Total yeasts and molds count	5.07	4.93	4.31	4.15	4.39	4.33	4.65	4.83	4.42	4.52	0.16	0.137
Lactic acid bacteria	5.37^de^	5.26^e^	6.01^c^	6.75^a^	5.38^de^	5.61^d^	6.33^b^	6.24^bc^	6.52^ab^	6.46^ab^	0.10	< 0.001
*B. subtilis*	5.75 ^d^	5.66^d^	7.82^a^	7.86^a^	5.92^d^	5.87^d^	7.51^ab^	7.10^bc^	7.71^a^	6.95^c^	0.17	< 0.001
Total coliform	6.94^a^	6.89^b^	6.53^g^	6.38^h^	6.62^fg^	6.56^g^	6.71^d^	6.78^c^	6.59^gh^	6.65^e^	0.03	< 0.001
*E. coli*	5.63^a^	5.57^a^	4.67^g^	4.47^h^	5.06^e^	4.86^f^	5.35^c^	5.45^b^	5.16^d^	5.22^d^	0.07	< 0.001
*Enterococcus* spp.	6.19^a^	5.95^b^	4.92^h^	4.81^i^	5.22^g^	5.35^f^	5.75^d^	5.85^c^	5.46^e^	5.69^d^	0.08	< 0.001

Means in a row with different superscripts are significantly different (P < 0.05).

^1^NC, non-injected group; PC, sterile distilled water; BS1 = Bacillus subtilis 4 × 10^5^/egg; BS2 = Bacillus subtilis 4 × 10^6^/egg; R1 = raffinose 2 mg/egg; R2 = raffinose 3 mg/egg; BS1R1 = (Bacillus subtilis 4 × 10^5^ + raffinose 2 mg)/egg; BS1R2 = (Bacillus subtilis 4 × 10^5^ + raffinose 3 mg)/egg; BS2R1 = (Bacillus subtilis 4 × 10^6^ + raffinose 2 mg)/egg; BS2R2 = (Bacillus subtilis 4 × 10^6^ + raffinose 3 mg)/egg. SEM, standard error of means.

There was a mistake in Table 6 as published. The decimal points in the values listed in the row titled “Surface area (μm^2^)” were incorrectly placed, shifting each value by one order of magnitude. The corrected table appears below.

**Table 6:** Effect of *in ovo* inclusion of *Bacillus subtilis*, raffinose, and their synbiotics on ileal morphology of 21-day old broilers.

Items (μm)	Treatment groups^1^										SEM	*P-*value
	NC	PC	BS1	BS2	R1	R2	BS1R1	BS1R2	BS2R1	BS2R2		
Villus height	717.48^cd^	522.61^e^	888.82^ab^	760.30^bc^	710.35^cd^	915.98^a^	607.11^de^	700.38^cd^	661.33^cde^	896.25^ab^	27.16	< 0.001
Villus width	92.48	119.91	101.38	86.77	128.68	101.37	125.07	118	123.86	121.84	4.222	0.239
Crypt depth	87.03	65.58	85.8	102.7	69.73	93.81	117.33	81.86	91.5	120.4	4.713	0.121
Muscularis thickness	203.37^b^	123.05^c^	172.89^bc^	182.31^bc^	126.81^c^	142.48^c^	181.03^bc^	201.74^b^	220.17^ab^	280.73^a^	11.23	0.029
Surface area (μm^2^)	207050.00	196670.00	285050.00	207310.00	290910.00	290210.00	235530.00	260080.00	257290.00	343040.00	32240.00	0.083

Means in a row with different superscripts are significantly different (P < 0.05).

^1^NC, non-injected group; PC, sterile distilled water; BS1 = Bacillus subtilis 4 × 10^5^/egg; BS2 = Bacillus subtilis 4 × 10^6^/egg; R1, raffinose 2 mg/egg; R2, raffinose 3 mg/egg; BS1R1 = (Bacillus subtilis 4 × 10^5^ + raffinose 2 mg)/egg; BS1R2 = (Bacillus subtilis 4 × 10^5^ + raffinose 3 mg)/egg; BS2R1 = (Bacillus subtilis 4 × 10^6^ + raffinose 2 mg)/egg; BS2R2 = (Bacillus subtilis 4 × 10^6^ + raffinose 3 mg)/egg. SEM, standard error of means.

In the published article, Supplementary Table 1, containing the Raw Cq/Ct values, was mistakenly omitted. “Table 1” has now been published alongside the original article.

Details pertaining to the treatment replicates, and the study design, were mistakenly omitted from **Materials and methods**, *Sample collection*, Paragraph 1. The corrected passage reads:

“On day 21, six birds per treatment (one bird/replicate) were randomly selected (to represent all treatment replicates) and euthanized by cervical dislocation. The current study was designed as a cross-sectional terminal evaluation at the conclusion of the starter and grower phases (21 days). Cecal contents were immediately collected into sterile tubes and stored at −20 °C for the microbial count and volatile fatty acid analysis. Ileal samples (approximately 1.5 cm in length of the mid-ileum) were excised and flushed with 0.9% saline to remove all the contents and then fixed in 10% buffered formalin solution for subsequent histomorphological investigations. A section of the mid-ileum (approximately 1.5 cm) was collected, washed with PBS, and immersed in Trizol reagent for subsequent gene expression investigation.”

“*B. subtills*” was used instead of “*B. subtilis*” in **Materials and Methods**, *Bacteriological Examination*, Paragraph 1. The corrected passage reads:

“Violet red bile agar, MacConkey agar, and *Bacillus* cereus agar (Oxoid) were used for total coliforms, *Escherichia coli*, and *B. subtilis*, respectively, after incubation at 37°C for 24 h. *Bacillus* cereus agar (Oxoid) was used for counting *B. subtilis* after incubation for 24 h at 37°C. MRS medium and Chromocult enterococci agar were used for lactic acid bacteria and *Enterococcus* spp., respectively, after incubation at 37°C for 48 h. The microbial counts were converted into log_10_ CFU g^−1^.”

Details regarding the validation of qPCR tests were mistakenly omitted from **Materials and methods**, *Quantitative Real-Time PCR analyses*, Paragraph 1. The corrected passage reads:

“The PCR cycling conditions included an initial denaturation at 95 °C for 12 min followed by 40 cycles of denaturation at 95 °C for 20 s, annealing at 60 °C for 30 s, and extension at 72 °C for 30 s. All qPCR tests were validated using melting curve analysis, which confirmed a single, distinct peak for each target gene, including β-actin, thereby ensuring high primer specificity and the absence of nonspecific amplification or primer dimers.”

A correction has been made to the section **Materials and methods**, *Statistical analysis*, Paragraph 1 to include details regarding how the study data were analyzed: “Data were analyzed according to a preplanned experimental set-up using one-way ANOVA (SPSS Inc., 2018).”

The “Kolmogorov–Smirnov test” was mistakenly referenced instead of the “Shapiro–Wilk normality test” in **Materials and methods**, *Statistical analysis*, Paragraph 3. Additionally, the corrected paragraph expands on the experimental unit structure by specifying the parameters measured per cage (BW, FI, FCR), clarifying the sampling approach and adding a justification for the statistical design choices regarding variance structure, nesting effects, and Type I error control. The corrected passage reads:

“Normality of the distribution was tested with the Shapiro–Wilk normality test, while the homogeneity of variance in the samples was assessed with Levene's test. The means were compared using Tukey multiple range test. The cage (replicate) served as the experimental unit for comparing growth performance (BW, FI, FCR) (*n* = 6 per treatment), while individuals' data served as the experimental units for the remaining parameters. One bird was randomly selected per replicate (*n* = 6 per treatment to represent all treatment replicates) to ensure biological diversity. Because only one bird was sampled per cage (replicate), there was no nesting of multiple birds within a single replicate for these specific measurements, which simplifies the variance structure. We acknowledge that the absence of a mixed model for nesting effects represents a simplified variance structure; however, birds were randomly selected from separate replicates to maintain biological independence and minimize potential unstable inference. This approach ensures that the degrees of freedom are correctly partitioned and that Type I error is not inflated by treating individual birds within a cage as independent observations due to the use of Tukey (HSD) for means comparison. Data are presented as means ± SEM, and the two significance was declared at *P* < 0.05.”

The original version of this article has been updated.

